# Process Intensification of 2,2′-(4-Nitrophenyl) Dipyrromethane Synthesis with a SO_3_H-Functionalized Ionic Liquid Catalyst in Pickering-Emulsion-Based Packed-Bed Microreactors

**DOI:** 10.3390/mi12070796

**Published:** 2021-07-05

**Authors:** Hong Zhang, Minjing Shang, Yuchao Zhao, Yuanhai Su

**Affiliations:** 1 Frontiers Science Center for Transformative Molecules, School of Chemistry and Chemical Engineering, Shanghai Jiao Tong University, Shanghai 200240, China; zhanghongsjtu@sjtu.edu.cn (H.Z.); mshang@sjtu.edu.cn (M.S.); 2 College of Chemistry and Chemical Engineering, Yantai University, Yantai 264005, China; 3 Key Laboratory of Thin Film and Microfabrication (Ministry of Education), Shanghai Jiao Tong University, Shanghai 200240, China

**Keywords:** packed-bed microreactor, process intensification, Pickering emulsion, ionic liquid, dipyrromethane

## Abstract

A stable water-in-oil Pickering emulsion was fabricated with SO_3_H-functionalized ionic liquid and surface-modified silica nanoparticles and used for 2,2′-(4-nitrophenyl) dipyrromethane synthesis in a packed-bed microreactor, exhibiting high reaction activity and product selectivity. The compartmentalized water droplets of the Pickering emulsion had an excellent ability to confine the ionic liquid against loss under continuous-flow conditions, and the excellent durability of the catalytic system without a significant decrease in the reaction efficiency and selectivity was achieved. Compared with the reaction performance of a liquid–liquid slug-flow microreactor and batch reactor, the Pickering-emulsion-based catalytic system showed a higher specific interfacial area between the catalytic and reactant phases, benefiting the synthesis of 2,2′-(4-nitrophenyl) dipyrromethane and resulting in a higher yield (90%). This work indicated that an increase in the contact of reactants with catalytic aqueous solution in a Pickering-emulsion-based packed-bed microreactor can greatly enhance the synthetic process of dipyrromethane, giving an excellent yield of products and a short reaction time. It was revealed that Pickering-emulsion-based packed-bed microreactors with the use of ionic liquids as catalysts for interfacial catalysis have great application potential in the process of intensification of organic synthesis.

## 1. Introduction

Dipyrromethanes and their derivatives are important building blocks for many chemicals containing porphyrin and polypyrrole structures. 2,2′-(4-nitrophenyl) dipyrromethane, a typical type of dipyrromethane, has been widely used in various fields such as materials science, medicine, optics, etc. [1,2,3,4,5]. Traditionally, 2,2′-(4-nitrophenyl) dipyrromethane is synthesized by the condensation of aldehydes with pyrroles via a double Friedel–Crafts reaction with acidic catalysts. Because of its importance, various homogeneous and heterogeneous catalysts have been exploited for the synthetic process of 2,2′-(4-nitrophenyl) dipyrromethane. For example, acetic acid [6], boric acid [7], and hydrochloric acid [8] were applied as homogeneous catalysts in aqueous phases for 2,2′-(4-nitrophenyl) dipyrromethane synthesis. However, defects such as low catalytic activity, a long reaction time, complicated post-treatment, environmental pollution, and reactor corrosion are still major challenges for these homogeneous catalytic systems. Taking into account the advantages of heterogeneous catalysts, such as easy separation and recycling, silica-supported sulfuric acid (H_2_SO_4_@SiO_2_) has been used for 2,2′-(4-nitrophenyl) dipyrromethane synthesis [9]. However, the tendency of deactivation by the absorption of generated impurities on the catalyst surface during the reaction, the loss of active ingredients by the regeneration treatment process, and the insufficient contact between the catalytic and liquid phases led to low catalytic activity, which all limited the heterogeneous catalyst’s practical application. Given this, it is essential to develop a catalyst with high activity, easy separation, and reuse for the synthesis of 2,2′-(4-nitrophenyl) dipyrromethane.

Recently, acidic task-specific ionic liquids (TSILs) have been widely used as environmentally friendly catalysts for 2,2′-(4-nitrophenyl) dipyrromethane synthesis [10,11,12,13,14]. In particular, SO_3_H-functionalized ionic liquids have become somewhat important catalysts and exhibited great potential in the replacement of conventional homogeneous and heterogeneous catalysts such as acetic acid, HCl, trifluoroacetic acid, acid cation exchange resin, and H_2_SO_4_@SiO_2_ [6,8,9,15,16,17]. Because it combines the advantageous characteristics of solid acids and mineral acids, it belongs to a “greener” and halogen-free ionic liquid that involves phosphate or octyloxy sulfate anions. Additionally, it is miscible with water, water tolerant, and suitable for reactions that need to proceed in water [18]. Some reports have proved that this type of catalyst has high catalytic activity in dipyrromethane synthesis. For instance, imidazole-based zwitterionic salts under solvent-free conditions and SO_3_H-IL ([bsmim][HSO_4_]) in the aqueous phase were adopted as homogeneous catalysts in this process by Chatterjee et al. and Senapak et al. [11,13], respectively, and both showed excellent catalytic activity. Although these ionic liquids have been proven with high catalytic activity, the requirement of a long reaction time and difficulty in the recovery and reuse of ionic liquids after reaction for dipyrromethane synthesis are still challenges. In particular, the high viscosity and harsh post-treatment conditions of ILs usually bring difficulties in separation and regeneration and approximately a 10% loss in the recycling process of ILs with high viscosities. Moreover, slow mass transfer in the reaction processes with the traditional operation mode and ineffective recycling are still hindering the large-scale use of ILs in industrial applications.

Pickering emulsion is a thermodynamically stable droplet system that is composed of solid nanoparticles adsorbed at the oil–water interface to form a layered or multilayered interface membrane without droplet aggregation [19,20,21,22]. By using the confined effect of emulsion droplets, limiting ionic liquid catalysts inside the droplets can prevent their loss. The catalytic efficiency based on the Pickering emulsion can be significantly improved because of the large interfacial area of the dispersed phase [23,24,25]. In recent years, Yang et al. made significant efforts in confined catalysis based on the Pickering emulsion, and they demonstrated that the application of the Pickering emulsion in sulfuric acid catalytic reactions showed great application potential in organic catalysis [23]. Packed-bed microreactors are a kind of microreactor widely applied in both homogeneous and heterogeneous catalytic processes, such as catalytic asymmetric synthesis, hydrogenation reactions, metal-catalyzed reactions, enzymatic catalysis, and photocatalysis [26,27,28,29]. Catalysts or inert microparticles are used as packing materials in packed-bed microreactors, and various reactants can mix acutely in the confined and crooked interstices between the microparticles, leading to the significant intensification of transport and reaction processes compared with nonpacked microchannels [30,31,32,33]. Furthermore, considering its advantages, the Pickering emulsion can be applied for the fabrication of packed-bed microreactors, in which stable and immobile droplets act as the packing materials and confine homogeneous catalysis efficiently. More importantly, continuous-flow processing with Pickering-emulsion-based packed-bed microreactors can facilitate the production of products without the post-treatment of ionic liquids. In particular, it can greatly enhance the mass transport and improve the catalytic efficiency simultaneously. 

In this work, a Pickering-emulsion-based catalytic system was used for the continuous synthesis of 2,2′-(4-nitrophenyl) dipyrromethane in packed-bed microreactors. SO_3_H-functionalized ionic liquid and surface-modified silica nanoparticles were synthesized and used for the construction of stable Pickering emulsion, and the important factors influencing the stability of the Pickering emulsion were explored. Then, the Pickering emulsion was filled into a microchannel to fabricate a packed-bed microreactor for the continuous synthesis of 2,2′-(4-nitrophenyl) dipyrromethane. The SO_3_H-functionalized ionic liquid acted as an efficient catalyst and was confined inside the Pickering emulsion droplets without loss for a long operation time. 2,2′-(4-nitrophenyl) dipyrromethane synthesis in the Pickering-emulsion-based packed-bed microreactor was compared with the liquid–liquid slug-flow microreactor and the batch reactor. Moreover, the catalytic excellence of the Pickering-emulsion-based packed-bed microreactor was revealed to correlate with the hydrodynamics. 

## 2. Experimental Section

### 2.1. Chemicals

Toluene, acetonitrile, ethyl alcohol, and pyrrole were purchased from Titan Technology Co., Ltd. (Shanghai, China). p-Nitrobenzaldehyde and tetraethyl orthosilicate (TEOS) were obtained from Sinopharm Reagent Co., Ltd. (Shanghai, China). Triethylamine, methyltrimethoxysilane (MTS), and sulfuric acid (98%) were purchased from Macklin Biochemical Co., Ltd. (Shanghai, China). Hydrochloric acid and 1,3-propane sultone of analytical grade were purchased from Sigma-Aldrich.

### 2.2. Equipment and Analysis Method

Proton nuclear magnetic resonance (^1^H NMR) (Bruker 400, AVANCE III HD 400, Karlsruhe, Germany) was used to analyze the ionic liquid structure. An FT-IR spectrometer (Spectrum 100, PerkinElmer Inc., Waltham, MA, USA) was applied to determine the successful modification of SiO_2_, and a scanning electron microscope (SEM, Nova Nano SEM 450, FEI Company, Hillsboro, ON, USA) was applied to characterize the microstructure of nanoparticles. The contact angle was investigated with an optical instrument (DSA30, Kruss Company, Hamburg, Germany) to quantify the hydrophobicity or hydrophilicity of the modified particles. Intermittent high-shear equipment (FA25, Fluko, Shanghai, China) was used to disperse the aqueous phase into the continuous phase to form a water-in-oil (W/O)-type Pickering emulsion. The concentrations of dipyrromethane and p-nitrobenzaldehyde in the mixture after reaction were measured by HPLC with a WondaSil C18-WR column (4.6 mm × 250 mm) and an ultraviolet–visible (UV–Vis) detector (SPDM20A, Shimazu, Tokyo, Japan) at a characteristic wavelength peak of 254 nm. The sample was injected into the column (equilibrated at 308 K) at the flow rate of 1.0 mL/min with MeOH/H_2_O (20/80 wt %) as the mobile phase. A high-speed CCD camera (PhantomLab110-12G, Wayne, NJ, USA) installed with a stereomicroscope (SZ2-CLS, Olympus, Tokyo, Japan) was used to capture the microstructure of the Pickering emulsion and the snapshots of slug flow inside the capillary microreactor.

### 2.3. Materials Synthesis

#### 2.3.1. Synthesis of 3-Triethylammonium Propane Sulfonic Hydrogen Sulfate

The synthetic procedure for 3-triethylammonium propane sulfonic hydrogen sulfate ([TEAPS][HSO_4_]) was as follows. Triethylamine and 1,3-propane sultone were mixed at a molar ratio of 1.2:1 in the solvent acetonitrile inside a 250 mL flask, and then the mixture was magnetically stirred at 80 °C for 20 min. After that, the intermediate product of 3-triethylammonium propane sulfonate (TEAPS) was collected by filtering and washed with acetonitrile three times. The pure TEAPS was dried at 40 °C for 12 h in a vacuum oven. Subsequently, a certain amount of TEAPS was dissolved in water, and then a certain volume of sulfuric acid was dropwise added into this aqueous phase. Next, this aqueous mixture was heated and stirred at 80 °C for 15 h. After the reaction, water was removed from the aqueous mixture at reduced pressure at 90 °C for 2 h, and the final product was obtained by washing with toluene and drying in a vacuum oven for 12 h.

#### 2.3.2. Synthesis of Surface-Modified Silica Nanoparticles

The silica (SiO_2_) nanoparticles were synthesized according to the Stöber method [34]. A certain amount of ammonium hydroxide, water, and ethyl alcohol was added into a flask and stirred at room temperature (25 °C) for 30 min. Then, a certain amount of TEOS was added into the mixture, and this reactive mixture was continuously stirred at different reaction times of 4 h, 8 h, and 16 h. After that, the SiO_2_ particles were collected by centrifugation and then washed four times with ethanol. After drying at 60 °C for 12 h, the obtained SiO_2_ particles were grafted by MTS [35]. The detailed surface modification procedure was as follows. Firstly, SiO_2_ nanoparticles were dispersed into toluene, and then an excessive amount of MTS was added, and the resulting solution was stirred for 4 h under nitrogen atmosphere at a temperature of 110 °C. The nanoparticles were isolated from the mixture by centrifugation and then washed repeatedly with toluene several times. Finally, the modified SiO_2_ nanoparticles were dried at 80 °C under vacuum for 24 h. According to various synthetic times, the surface-modified SiO_2_ nanoparticles were named MTS@SiO_2_ (4 h), MTS@SiO_2_ (8 h), and MTS@SiO_2_ (16 h).

#### 2.3.3. Synthesis of the Ionic Liquid-Based Pickering Emulsion

The preparation of the Pickering emulsion was realized as follows. A certain amount of TEAPS was dissolved in water as the dispersed phase, and then this dispersed phase together with MTS@SiO_2_ was dispersed in toluene under ultrasound. Afterward, the mixture was added into a flask and stirred by a high-shear mixer at 10,000 rpm for 2 min to obtain a viscous and milky emulsion. After synthesis of the Pickering emulsion, a drop of the emulsion was taken out immediately and then dripped onto a piece of a clean glass slide to characterize the microstructure of the Pickering emulsion.

#### 2.3.4. Synthesis of 2,2′-(4-Nitrophenyl) Dipyrromethane in Different Reactors

**Figure 1:**
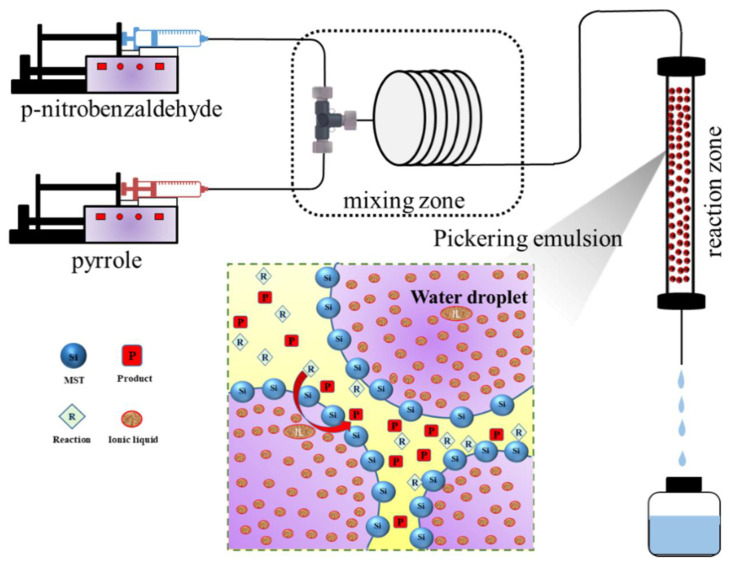
Schematic diagram for the continuous synthesis of 2,2′-(4-nitrophenyl) dipyrromethane in the Pickering-emulsion-based packed-bed microreactor.

The stable Pickering emulsion was transferred into a stainless-steel tube to fabricate the packed-bed microreactor. This tube had an inner diameter of 4.6 mm and a length of 15 cm with a filtering net placed as the supporter in the bottom. For the construction of this Pickering-emulsion-based packed-bed microreactor, the stable Pickering emulsion was slowly filled into the tube by hanging vertically. After that, both the inlet and outlet of the tubular packed-bed microreactor were connected with fittings. As shown in Figure 1, prior to the catalytic Friedel–Crafts alkylation in the Pickering-emulsion-based packed-bed microreactor, a piece of perfluoroalkoxy capillary (PFA, IDEX Health & Science LLC., Sacramento, CA, USA) was applied to mix the reactants of pyrrole and p-nitrobenzaldehyde. The concentrations of pyrrole and p-nitrobenzaldehyde in the two reactant solutions were 2.4 mmol/L and 1.0 mmol/L, respectively. These two reactant solutions were introduced into the capillary through a T-micromixer by two spring pumps (New Era Pump System, Farmingdale, NY, USA) by maintaining the total volumetric flow rate of 0.02 mL/min. The outlet of the Pickering-emulsion-based packed-bed microreactor was collected to a second capillary for sample collection.

**Figure 2:**
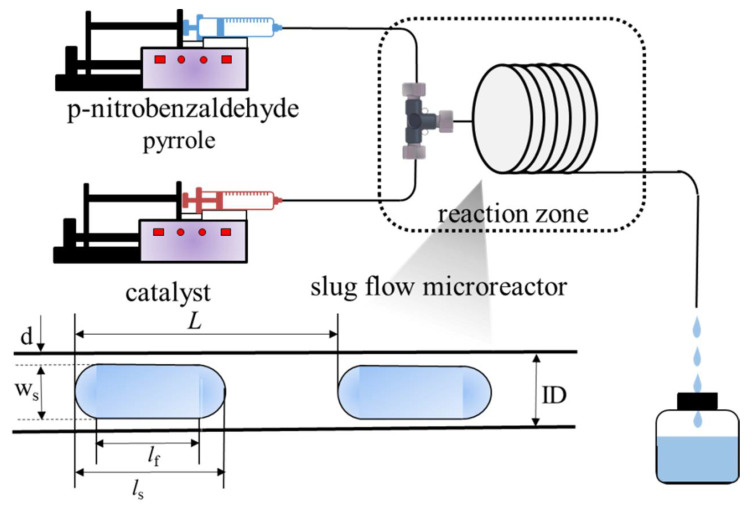
Schematic diagram for the continuous synthesis of 2,2′-(4-nitrophenyl) dipyrromethane in the liquid–liquid slug-flow microreactor.

The schematic diagram of the 2,2′-(4-nitrophenyl) dipyrromethane synthesis process in the liquid–liquid slug-flow microreactor is shown in Figure 2. The solutions of reactants (the concentrations of pyrrole and p-nitrobenzaldehyde were, respectively, 1.2 mmol/L and 0.5 mmol/L, and the catalyst ([TEAPS][HSO_4_] concentration was 33.1 wt %, which was related to the mass fraction of the dispersed phase) were delivered into the capillary microreactor system by two syringe pumps. This microreactor system was mainly constructed with a T-micromixer (ID, 1.0 mm) and PFA capillary with the same inner diameter (ID, 1.0 mm). Both the T-micromixer and the capillary were submerged in a water bath to maintain the set temperature (30 °C). The volumetric flow rates of the reactant and catalyst solutions were adjusted with the variation of the capillary length to maintain the same residence time. After the reaction, the reactant and catalyst phases were immediately separated in a collector, and the reactant phase was collected as soon as possible to analyze the yield of products and the conversion of reactants by HPLC.

For the dipyrromethane synthesis in the batch reactor, the reaction was carried out in a 50 mL bottom flask with magnetic stirring at 500 rpm and a temperature of 30 °C, and the concentrations of reactants and the catalyst were the same as the above-mentioned values.

## 3. Result and Discussion

### 3.1. Characterization of the [TEAPS][HSO_4_] Ionic Liquid and Surface-Modified SiO_2_ Nanoparticles

**Figure 3:**
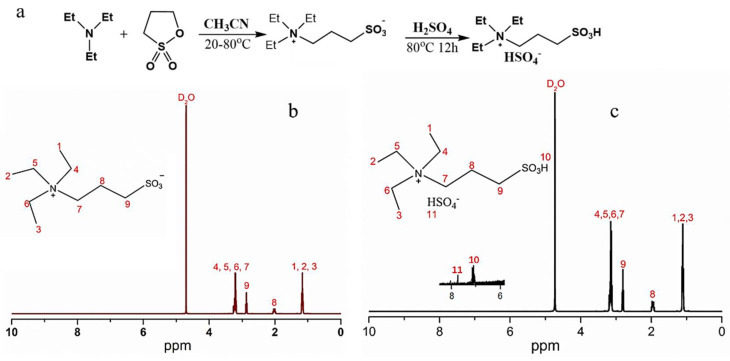
Synthetic route of the [TEAPS][HSO_4_] ionic liquid (**a**), proton nuclear magnetic resonance (^1^H NMR) spectra of the TEAPS intermediate compound (**b**), and the final product of the [TEAPS][HSO_4_] ionic liquid (**c**).

The Pickering emulsion was fabricated with the [TEAPS][HSO_4_] ionic liquid in droplets in the dispersed phase and hydrophobic silica nanospheres as a solid emulsifier. According to a two-step method, the [TEAPS][HSO_4_] ionic liquid was successfully synthesized using inexpensive materials, as presented in Figure 3a [14]. The structures of the TEAPS intermediate compound and the final synthesized [TEAPS][HSO_4_] were confirmed by ^1^H NMR spectra (see Figure 3b,c), demonstrating the obtained intermediate and product with high purity.

**Figure 4:**
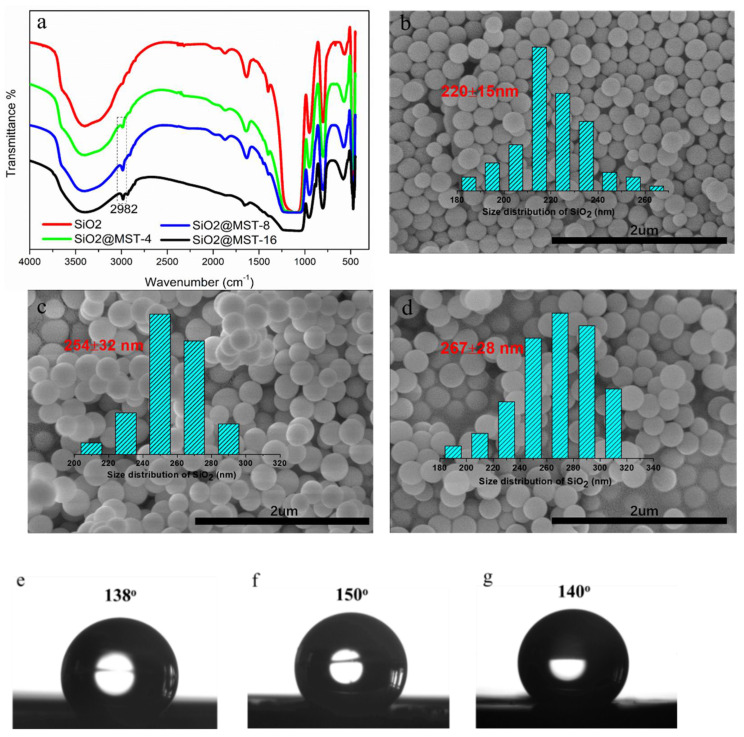
(**a**) FT-IR spectra of SiO_2_ and surface-modified SiO_2_, (**b**–**d**) SEM images of different surface-modified SiO_2_, and (**e**–**g**) contact angles of different surface-modified SiO_2_ nanoparticles.

The surface-modified SiO_2_ nanoparticles were characterized by FT-IR spectra, SEM images, and contact angles, as shown in Figure 4. It can be seen that the FT-IR spectra of the surface-modified SiO_2_ nanoparticles present a new absorption peak at the wavelength of 2982 cm^–1^ belonging to the C-H stretching vibration, compared to the FT-IR spectra of the SiO_2_ nanoparticles without the surface modification (Figure 4a). This demonstrated that a large number of methyl groups were grafted onto the surface of SiO_2_ nanoparticles. The contact angle of SiO_2_ without the surface modification was determined to be 16° according to the reported literature, indicating its hydrophilicity [36], while the contact angles of the surface-modified SiO_2_ nanoparticles were, respectively, determined as values of 138°, 150°, and 140° (Figure 4e–g), which were larger than 90°. These results consistently confirmed that the SiO_2_ nanoparticles were successfully modified by MTS with hydrophobic properties. The average sizes of these MTS@ SiO_2_ nanoparticles were calculated to be 220 nm, 254 nm, and 267 nm, respectively, as presented in Figure 4b–d.

### 3.2. Stability of the Ionic-Liquid-Based Pickering Emulsion

**Figure 5:**
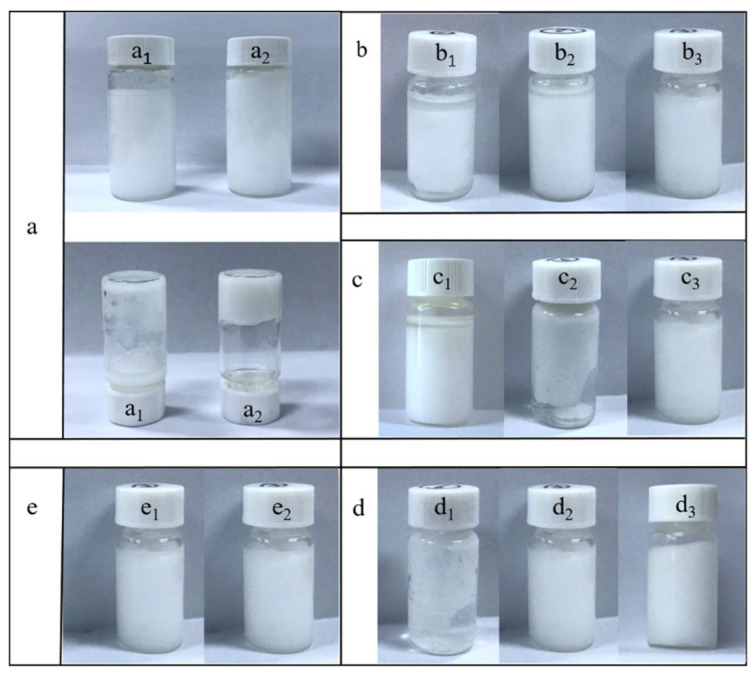
Stability of the Pickering emulsion under different synthetic conditions: (**a**) effects of the solid concentration (**a_1_**: 3.75 wt % and **a_2_**: 7.5 wt %) on the mobility of the Pickering emulsion; (**b**) different ionic liquid concentrations (**b_1_**: 3.5 wt %, **b_2_**: 13.3 wt %, and **b_3_**: 33.1 wt %); (**c**) volumetric ratio of the aqueous phase to the oil phase (**c_1_**: 1:1, **c_2_**: 1:2.7, and **c_3_**: 1:2); (**d**) different surface-modified SiO_2_ nanoparticles (**d_1_**: MTS@SiO_2_ (4 h), **d_2_**: MTS@SiO_2_ (8 h), and **d_3_**: MTS@SiO_2_ (16 h)); (**e**) stability of the Pickering emulsion (**e_1_**: 14 days and **e_2_**: 30 days).

The long-term stability of the Pickering emulsion is of vital importance for its application in the field of organic synthesis. Figure 5 presents some key factors that affect the stability of the Pickering emulsion. As can be seen in Figure 5(a_1_), the emulsification was achieved using MTS@SiO_2_ as the Pickering surfactant at a concentration of 3.75 wt %, but this Pickering emulsion could not be used for dipyrromethane synthesis because of the lack of stability. After increasing the MTS@SiO_2_ content to 7.5 wt %, the Pickering emulsion became much more stable and had poor fluidity and strong immobility (Figure 5(a_2_)). This was mainly due to the increased number of solid particles leading to smaller droplets and a larger coverage area by the nanoparticles. As for the effect of the ionic liquid concentration, the aqueous-phase droplets would be quickly aggregated and then layered from the organic phase if the Pickering emulsion was formed at a relatively low concentration of ionic liquid (see Figure 5(b_1_,b_2_)). The increasing amount and viscosity of ionic liquid were beneficial for stabilizing the Pickering emulsion (see Figure 5(b_3_)), which made the dispersed droplets more rigid than the pure aqueous droplets. The viscosity of the [TEAPS][HSO_4_] ionic liquid was tested with a value of 7000 cP, which was an order of magnitude higher than that of conventional ionic liquids. The proper volumetric ratio of water and oil was optimized at a value of 2.0, as shown in Figure 5c. Interestingly, the synthetic time of MTS@SiO_2_ had a crucial influence on the stability of the Pickering emulsion (Figure 5d). The surface modification time of SiO_2_ nanoparticles should be long enough to ensure that the synthesized nanoparticles are stably absorbed at the oil–water interface in the Pickering emulsion. The conditions for obtaining a stable Pickering emulsion were achieved; that is, a solid content of 7.5%, an ionic liquid content of 33.6%, an oil-to-water volumetric ratio of 1:2, and a modified silica particle size of 254 nm were selected rationally. The surface-modified SiO_2_ nanoparticles were helpful in stabilizing the W/O Pickering emulsion and promoted the adsorption of organic substrates at the interface between the aqueous and organic phases in the Pickering emulsion [37,38]. The Pickering emulsion formulated under optimal conditions was obtained as a milky substance and was fully resistant against destabilization phenomena, such as coalescence, creaming, and sedimentation. After keeping the synthesized Pickering emulsion for two weeks or even one month, the emulsion was still very stable (see Figure 5e).

**Figure 6:**
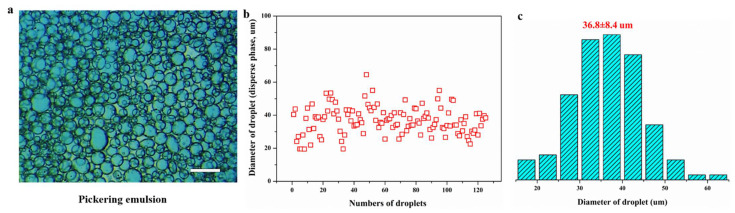
(**a**) Microscopic picture captured immediately after the formation of the stable Pickering emulsion (the measurement scale was 100 μm), (**b**) the calculated diameter of droplets (dispersed phase), and (**c**) its size distribution.

**Figure 7:**
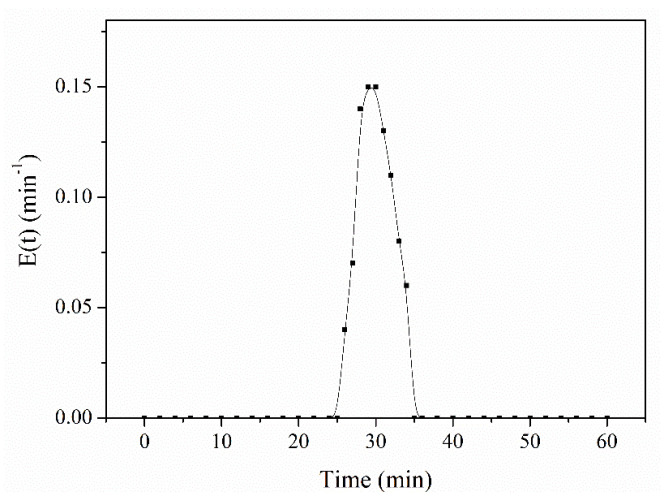
Residence time distribution in the Pickering-emulsion-based packed-bed microreactor.

Images of the dispersed droplets were recorded by an optical microscope, as shown in Figure 6. These images confirmed that the Pickering emulsion belonged to the W/O type with dense and small aqueous droplets, as seen in Figure 6a. These results also demonstrated that the Pickering emulsion was more stable and more likely to form smaller emulsion droplets compared to conventional emulsion systems. More than 100 droplets were chosen for the calculation of the droplets’ diameter. It was observed that the narrower size distribution of the droplets’ diameter was obtained (Figure 6b). This was likely associated with the formation process of droplets involving the breakup and coalescence for the larger diameter of the emulsion droplets. The diameter of droplets (d_32_) varied between 20 μm and 80 μm. The mean size of droplets was 36.8 μm (see Figure 6b). Moreover, the pulse method was used with the tracer of Sudan Red to analyze the residence time distribution in the Pickering-emulsion-based packed-bed microreactor system. It was found that the residence time distribution curve was narrow, implying that the flow in the Pickering-emulsion-based packed-bed microreactor was very close to the plug flow, and the obtained average residence time was 30 min, as shown in Figure 7.

### 3.3. Reaction Performance of 2,2′-(4-Nitrophenyl) Dipyrromethane Synthesis in the Pickering-Emulsion-Based Packed-Bed Microreactor

**Figure 8:**
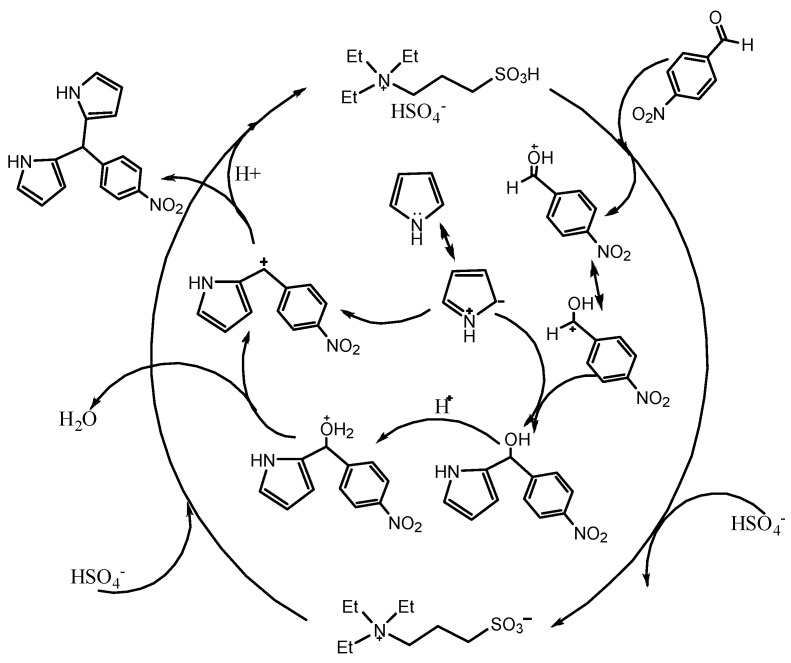
Mechanism of the 2,2′-(4-nitrophenyl) dipyrromethane synthesis with the use of the [TEAPS][HSO_4_] ionic liquid catalyst.

The synthesized stable Pickering emulsion was packed in the microreactor for 2,2′-(4-nitrophenyl) dipyrromethane synthesis. Before investigating the reaction performance in the Pickering emulsion system, we conducted a control experiment in which the [TEAPS][HSO_4_] ionic liquid was absent from the synthetic system. It was found that the Friedel–Crafts reaction hardly occurred. The SO_3_H-functionalized ionic liquid acted as a catalyst for the condensation of p-nitrobenzaldehyde with pyrrole via the double Friedel–Crafts reaction. Multistep Friedel–Crafts alkylation catalyzed by the Brønsted acid of [TEAPS][HSO_4_] is presented as a possible mechanism, as shown in Figure 8. The aromatic nucleophilic addition reaction takes place for p-nitrobenzaldehyde at the beginning. [TEAPS][HSO_4_] Brønsted acid provides hydrogen protons, which promotes electron transfer between the reactants. The arene intermediate compound containing the carbocation moiety immediately reacts with pyrrole via the nucleophilic attack. The 2,2′-(4-nitrophenyl) dipyrromethane is formed following a typical aromatic electrophilic substitution mechanism [39]. The aforementioned mechanism also proves the greenness of this process because equimolar water is produced during the synthesis process of dipyrromethane.

The [TEAPS][HSO_4_] ionic liquid catalyst is highly water-soluble and insoluble in toluene [40]. Pyrrole, p-nitrobenzaldehyde, and the product of 2,2′-(4-nitrophenyl) dipyrromethane are almost insoluble in water. Apparently, a reaction between pyrrole and carbonyl compounds may occur at the oil–water interface. The reactants of pyrrole and p-nitrobenzaldehyde reached the oil–water interface through diffusion and formed a product on the surface of the aqueous droplets containing the IL catalyst. The dipyrromethane was released from the aqueous layer as it was formed, and the produced water was in the aqueous phase, driving the reaction to completion, as shown in Figure 1.

**Figure 9:**
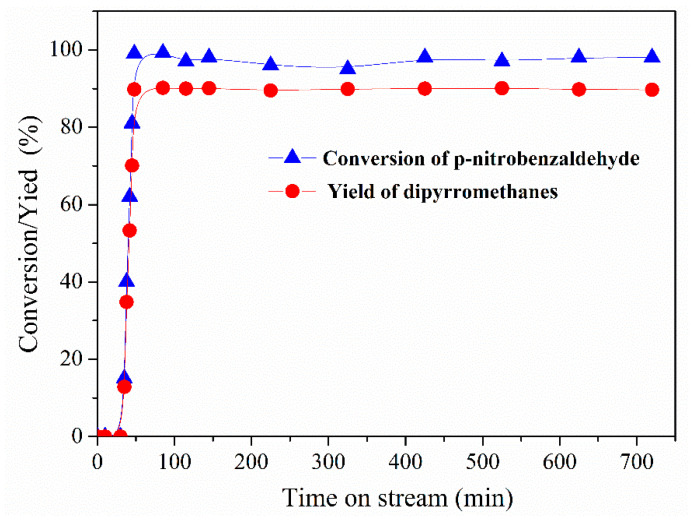
Conversion of p-nitrobenzaldehyde and the yield of 2,2′-(4-nitrophenyl) dipyrromethane in the Pickering-emulsion-based packed-bed microreactor.

A large specific interfacial area between the organic and aqueous (catalysis) phases in the Pickering emulsion was expected, leading to the fast conversion of reactants. The reaction performance was investigated in the Pickering-emulsion-based packed-bed microreactor. The p-nitrobenzaldehyde was quickly converted, and a high yield of dipyrromethane was obtained, as indicated in Figure 9. At a short operating time, the conversion of p-nitrobenzaldehyde rapidly increased to 100%. In the subsequent operating time, the conversion reached a plateau at 99–100% with slight fluctuations. The selectivity toward dipyrromethane was always maintained at approximately 90%. Such high yield and selectivity were comparable to those of reported homogeneous and heterogeneous catalytic systems [1,2,3,4,5,6,7,8,9,11,13]. Impressively, even after the running time was prolonged to more than 12 h, the conversion and yield did not show a significant decrease. These results confirm that compartmentalized aqueous droplets had an excellent ability to confine the ionic liquid against loss under continuous-flow conditions. Therefore, such a confined effect was responsible for the excellent durability without a significant decrease in the reaction efficiency and selectivity. Moreover, no IL catalyst was found in the collection bottle during the reaction, again proving the robustness of this confined catalysis system.

**Figure 10:**
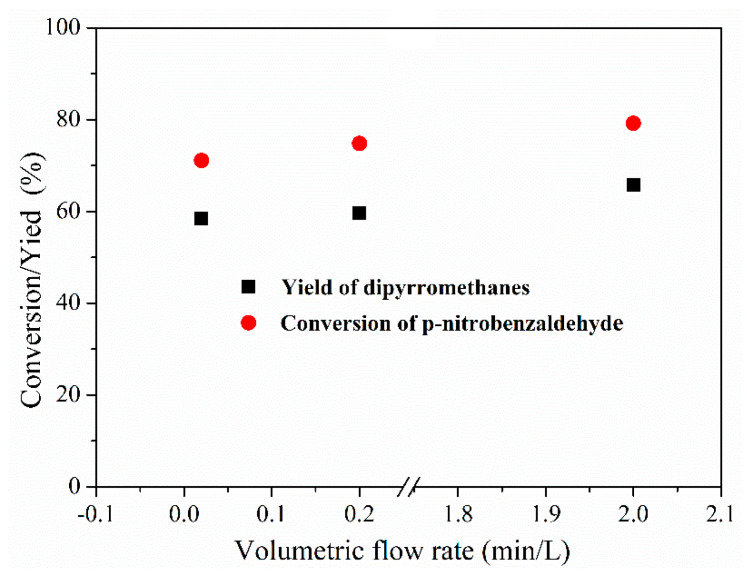
Conversion of p-nitrobenzaldehyde and the yield of 2,2′-(4-nitrophenyl) dipyrromethane in the liquid–liquid slug-flow microreactor.

To further evaluate the interfacial catalysis in the Pickering emulsion system, we compared it with the reaction performance in the liquid–liquid slug-flow microreactor and the batch reactor. The reaction conditions for dipyrromethane synthesis in the liquid–liquid slug-flow microreactor and the batch reactor were the same as those in the Pickering-emulsion-based packed-bed microreactor. To confirm the interfacial catalysis, we firstly examined the effect of the flow rate on the reaction performance of the 2,2′-(4-nitrophenyl) dipyrromethane synthesis in the slug-flow microreactor. Various interfacial areas were achieved by adjusting the flow rate. From the data of the conversion and yield against the flow rate in Figure 10, one can clearly see that the conversion of p-nitrobenzaldehyde and the yield of 2,2′-(4-nitrophenyl) dipyrromethane gradually increased with the increase in the flow rate in the slug-flow microreactor. The increase in the flow rate indicated a larger interface between the reactant and catalyst phases, which accelerated the mass transfer in the slug-flow microreactor leading to higher conversion of reactants and higher yield of product. The conversion of p-nitrobenzaldehyde and the yield of 2,2′-(4-nitrophenyl) dipyrromethane were obtained with values of 80% and 67%, respectively, at a flow rate of 2.0 mL/min.

**Figure 11:**
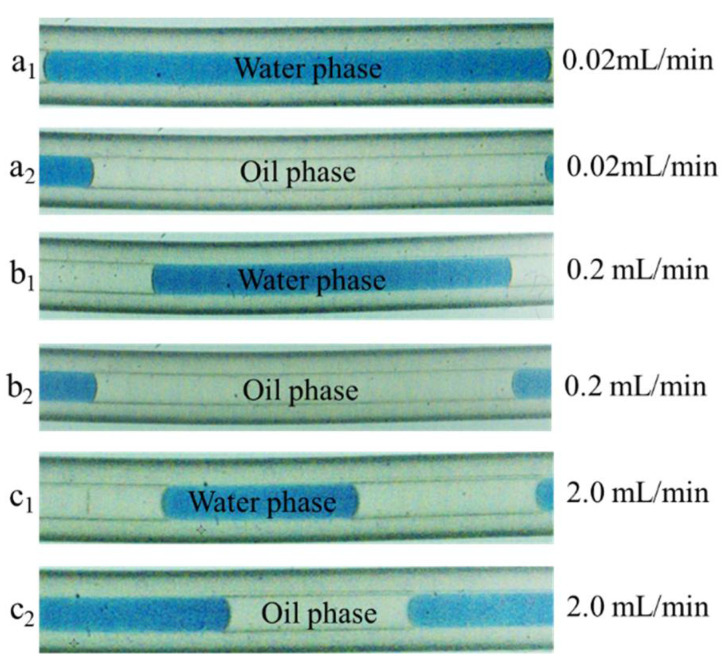
Images of toluene/water-phase slug flow formed in the capillary microreactor under different flow rates: 0.02 mL/min (**a_1_**,**a_2_**), 0.2 mL/min (**b_1_**,**b_2_**), and 2 mL/min (**c_1_**,**c_2_**).

Afterward, the specific interfacial area of the toluene/water/ionic liquid-formed slug-flow microreactor was investigated. The flow patterns of slug flow were captured through a CCD camera at different flow rates. Because the capillary microreactor was made of PFA (a hydrophobic material), the inner wall of the capillary microreactor was wetted by an organic thin film, and the aqueous droplets were dispersed in the organic phase separately. An alternating sequence of uniform slugs was formed at low flow rates from 0.02 to 2.0 mL/min [41]. Although the wall film between the capillary and slugs could not be observed within the resolution of the photography in Figure 11, it would be indeed presented due to the hydrophobic characteristics of the PFA material, and its thickness would be well below 25 μm [42]. The specific interfacial area of slug flow with an organic film was determined by the following equations [43,44]:

(1) $$\alpha_{1}=\frac{4\left(2w_{s}+l_{s}-l_{f}\right)\left(l_{s}-l_{f}\right)+w_{s}l_{f}}{w_{s}\left[3w_{s}^{}l_{f}+2\left(l_{s}-l_{f}\right)\right]\left(1+1/q\right)}$$

(2) $$d=1.34\;ID_{}Ca^{2/3}$$

(3) $$Ca=\frac{\mu_{M}U_{M}}{\gamma }$$

Moreover, the specific interfacial area in the Pickering-emulsion-based packed-bed microreactor was calculated according to the following equation [45]:

(4) $$\alpha_{w/o}=\frac{6\phi_{d}}{d_{32}}$$

where $\phi_{d}$ is the dispersed phase volume fraction.

**Figure 12:**
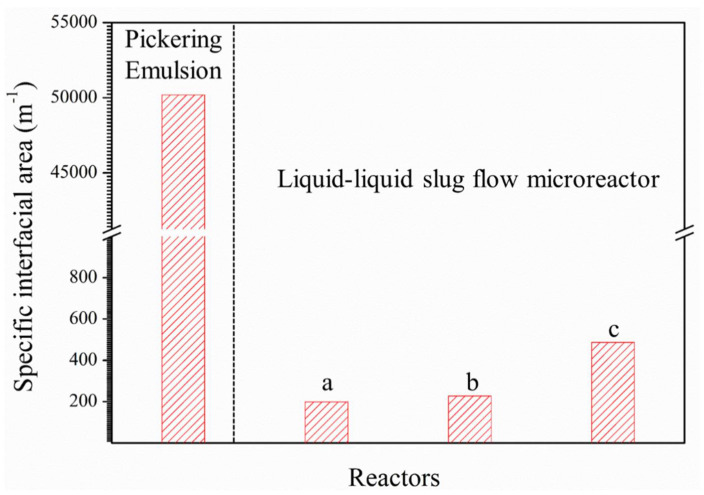
Calculated specific interfacial area of the dispersed phase in the Pickering emulsion and liquid–liquid slug-flow microreactors at different flow rates: (**a**) 0.02 mL/min, (**b**) 0.2 mL/min, and (**c**) 2.0 mL/min.

The specific interfacial area in the Pickering-emulsion-based packed-bed microreactor was compared with that in the liquid–liquid slug-flow microreactor [22], as shown in Figure 12. In the slug-flow microreactor, the specific interfacial area increased from 1.98 × 10^2^ to 4.87 × 10^2^ m^–1^ with the increase in the flow rate from 0.02 to 2.0 mL/min. The tendency of the specific interfacial area in the slug-flow microreactor confirmedly demonstrated the domination of the interfacial catalysis over the formation of dipyrromethane. 

The specific interfacial area in the Pickering-emulsion-based packed-bed microreactor was calculated with the value of 1.09 × 10^5^ m^–1^ according to Equation (4). It is well known that the droplets were always partially covered with nanoparticles for the stable Pickering emulsion. However, the surface coverage of nanoparticles should be considered for the calculation of the effective specific interfacial area; that is, the ratio of the coverage area (*C*) of nanoparticles adsorbed on the interface to the total droplet surface area should be taken into account [46], which is expressed by the following equation [47]:

(5) $$C=\frac{\phi_{p}R}{4\phi_{d}r}$$

where *r* and *R* are the average radii of nanoparticles and droplets, respectively. Therefore, the effective specific interfacial area ($\alpha_{w/o}{}^{\prime }$) can be calculated by the following equation:

(6) $$\alpha_{w/o}{}^{\prime }=\alpha_{w/o}(1-C)$$

The effective specific interfacial area in the Pickering-emulsion-based packed-bed microreactor was obtained with a value of 5.02 × 10^4^ m^–1^, which was two orders of magnitude higher compared to the liquid–liquid slug-flow microreactor. The silica nanoparticles were absorbed at the interface between the aqueous and organic phases to form the stable W/O emulsion, creating a large specific interfacial area for the contact and conversion of reactants with the effect of the IL catalyst. Moreover, the Pickering emulsion system could reduce the diffusion of the reactants into the interior of the aqueous droplets. The larger effective specific interfacial area and the shorter diffusion distance increased the mass transfer rate and resulted in higher conversion and higher yield in the Pickering-emulsion-based packed-bed microreactor compared to the slug-flow microreactor. For example, the obtained yield of dipyrromethane in the Pickering-emulsion-based packed-bed microreactor was about 23% higher than that in the slug-flow microreactor at the same reaction time of 30 min. 

**Figure 13:**
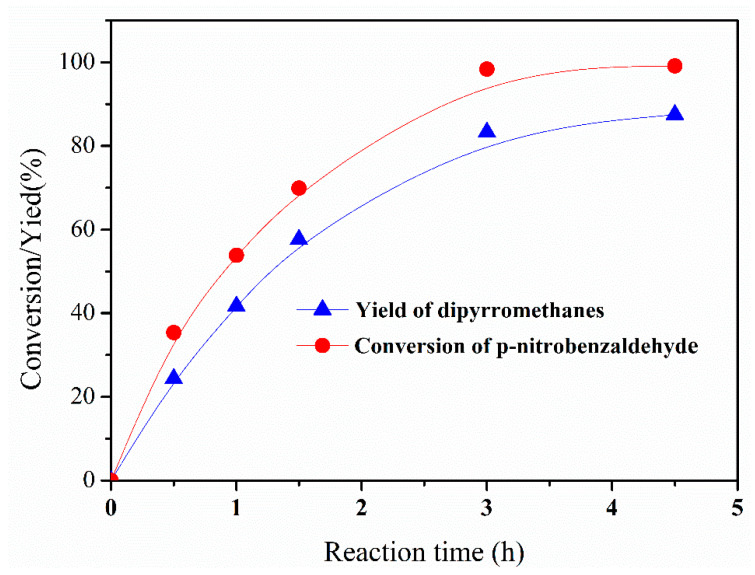
Conversion of p-nitrobenzaldehyde and the yield of 2,2′-(4-nitrophenyl) dipyrromethane in the batch reactor at various reaction times.

In the batch reactor without the use of the emulsifier, the conversion of p-nitrobenzaldehyde was about 35%, and the yield of 2,2′-(4-nitrophenyl) dipyrromethane reached about 24% at 30 min, which were much lower than those obtained in the Pickering-emulsion-based packed-bed microreactor and the slug-flow microreactor, as depicted in Figure 13. By prolonging the reaction time to 4.5 h, the conversion of p-nitrobenzaldehyde could reach about 100%, and the highest yield of 2,2′-(4-nitrophenyl) dipyrromethane was nearly 85%. Obviously, the synthetic process in the batch reactor would have a much longer reaction time and obtain lower selectivity and yield of 2,2′-(4-nitrophenyl) dipyrromethane compared to the Pickering-emulsion-based packed-bed microreactor. Additionally, in comparison with the other reported catalytic systems for 2,2′-(4-nitrophenyl) dipyrromethane synthesis in the batch reactors, the TEAPS-based catalytic system, in combination with the Pickering-emulsion-based packed-bed microreactor, was more efficient and environmentally friendly, as can be seen in Table 1.

The continuous-flow processing with the Pickering-emulsion-based packed-bed microreactor could ensure the products existing in the organic phase to be easily taken out from the catalytic system and protect the products from further reactions. In addition, such a packed-bed microreactor system confined the catalyst inside the dispersed droplets with immobilization, and its unique interfacial effect could intensify the mass transfer and greatly improve the reaction efficiency, overcoming the defects of both the batch reactor and the liquid–liquid slug-flow microreactor, such as a long reaction time, low product yield, and complex post-treatment of catalysts.

## 4. Conclusions

We demonstrated an efficient strategy to enhance 2,2′-(4-nitrophenyl) dipyrromethane synthesis with the use of a Pickering-emulsion-based packed-bed microreactor. Synthesized SO_3_H-functionalized ionic liquid as a catalyst and surface-modified silica nanoparticles as an emulsifier were applied to construct the Pickering emulsion. The effects of solid and ionic liquid contents, the oil-to-water volumetric ratio, and surface-modified nanoparticles on the stability of the Pickering emulsion were investigated. The stable Pickering emulsion was used to fabricate a packed-bed microreactor for the continuous synthesis of 2,2′-(4-nitrophenyl) dipyrromethane. 

The Pickering-emulsion-based packed-bed microreactor showed high catalytic efficiency and selectivity for 2,2′-(4-nitrophenyl) dipyrromethane synthesis, and the 2,2′-(4-nitrophenyl) dipyrromethane yield could reach 90%. It also exhibited a good confinement effect and high stability. The ionic liquid could be confined in the aqueous droplets against loss, and the reaction performance of this catalytic system remained constant during long-term operation under continuous- flow conditions.

Compared with the liquid–liquid slug-flow microreactor and the conventional batch system, the Pickering-emulsion-based packed-bed microreactor presented much higher reaction efficiency for 2,2′-(4-nitrophenyl) dipyrromethane synthesis. The reaction efficiency was found to be highly related to the effective specific interfacial area and the diffusion distance. Much smaller droplets from the confinement effect of the Pickering-emulsion-based packed-bed microreactor created a larger specific interfacial area for the sufficient contact of reagents, which resulted in a higher selectivity and yield for 2,2′-(4-nitrophenyl) dipyrromethane synthesis. In short, Pickering-emulsion-based packed-bed microreactors in combination with ionic liquids provide promising platforms for the process intensification of organic synthesis, especially for other types of dipyrromethane synthesis.

## Figures and Tables

**Table 1:** Comparison between the Pickering-emulsion-based packed-bed microreactor and the batch reactors for the synthesis of 2,2′-(4-nitrophenyl) dipyrromethane.

Reactors	Reaction Time	Catalytic Condition	Yield	Reference
Batch reactor	1 h	Acetic acid, n(pyrrole): n(aldehyde) = 2, solvent: THF/acetic acid = 9:1	35%	[6]
Batch reactor	15 min	Catalyst: hydrochloric acid, n(pyrrole): n(aldehyde) = 2, water solvent,	37%	[8]
Batch reactor	4 min	50 mg silica-supported sulfuric acid at room temperature, n(pyrrole): n(aldehyde) = 2	58%	[9]
Batch reactor	10–15 h	Acid cation exchange resin, n(pyrrole): n(aldehyde) = 20	61%	[15]
Batch reactor	_	Trifluoroacetic acid, V(pyrrole):V(aldehyde) = 25	56%	[16]
Batch reactor	8 min	Catalyst: sulfated polyborate, n(pyrrole): n(aldehyde) = 2, solvent-free	69%	[17].
Batch reactor	4.5 h	Catalyst: [TEAPS][HSO4], n(pyrrole): n(aldehyde) = 2.4	85%	This work
Pickering-emulsion-based packed-bed microreactor	30 min	Catalyst: [TEAPS][HSO4], n(pyrrole): n(aldehyde) = 2.4	90%	This work
